# New dawn of cellular therapies in autoimmune diseases

**DOI:** 10.1016/j.omtm.2023.101141

**Published:** 2023-11-08

**Authors:** Dimitrios Laurin Wagner, Lennard Ostendorf

**Affiliations:** 1 Berlin Center for Advanced Therapies, Charité – Universitätsmedizin Berlin, corporate member of Freie Universität Berlin, Humboldt-Universität zu Berlin, and Berlin Institute of Health, Berlin, Germany; 2 BIH Center for Regenerative Therapies, Berlin Institute of Health at Charité – Universitätsmedizin Berlin, Berlin, Germany; 3 Institute of Transfusion Medicine, Charité – Universitätsmedizin Berlin, corporate member of Freie Universität Berlin, Humboldt-Universität zu Berlin, and Berlin Institute of Health, Berlin, Germany; 4 Department of Nephrology and Medical Intensive Care, Charité – Universitätsmedizin Berlin, corporate member of Freie Universität Berlin, Humboldt-Universität zu Berlin, and Berlin Institute of Health, Berlin, Germany; 5 Deutsches Rheuma-Forschungszentrum (DRFZ), an Institute of the Leibniz Association, Berlin, Germany; 6 BIH Biomedical Innovation Academy, BIH Charité Junior Clinician Scientist Program, Berlin Institute of Health at Charité – Universitätsmedizin Berlin, Berlin, Germany

## Main text

In many autoimmune diseases, autoreactive antibodies contribute to the pathogenesis or directly induce organ dysfunction. These antibodies are secreted by B and plasma cells, which have thus been identified as important treatment targets. However, some antibody-secreting cells, such as long-lived plasma cells (LLPCs), have remained elusive targets. Their proliferative senescence precludes antiproliferative approaches and their negativity for CD20 precludes an attack with rituximab or other αCD20 monoclonal antibodies.^1^ Other B cells escape targeting by transiently downregulating CD20 or hiding in tissue niches.^2^

CD19-targeting chimeric antigen receptor (CAR) T cells now have shown remarkable short-term efficacy in a case series of patients with the autoimmune disease systemic lupus erythematosus (SLE) by depleting most autoantibody-secreting cells.^3^^,^^4^ The ability of this “living drug” to invade tissues likely leads to an increased treatment effect compared with monoclonal antibodies.

In this issue of *Molecular Therapy: Methods & Clinical Development*, Nunez et al.^5^ present data on proinflammatory cytokines, autoantibodies, and vaccine-induced protective antibodies before and 3 months after CD19-CAR T cell therapy, thereby expanding our understanding of the biological mode of action of CAR T cells in SLE. Although this approach has undoubtedly been very successful in the reported cases, a controlled trial and long-term outcome data are still pending.

The adaptation of CD19-CAR T cell-based therapies to target B and plasma cells is only the next step in a long history of treatment approaches of malignant diseases translated to autoimmune diseases. Previous examples include the depletion of pathogenic autoimmune B cells with the anti-CD20 antibody rituximab, targeting of LLPCs with the anti-CD38 antibody daratumumab,^6^ and autologous hematopoietic stem cell transplantation to “reset” the immune memory of both B and T cells.^1^

Anti-CD19 CAR T cells are likely only the beginning of wider and more targeted use of cellular therapies in autoimmune diseases: regulatory T cell products have been successfully used in early-stage clinical trials for organ transplantation and are especially interesting for diseases in which T-cell-mediated organ damage plays an important role.^7^^,^^8^ For autoantibody-mediated autoimmune diseases, CAR T cells targeting more specific plasma cell antigens (such as BCMA) are currently in clinical trials for autoimmune diseases. A recent census counted 14 ongoing trials using CD19 and/or BCMA-targeted CAR T cells in SLE alone.^9^

The “holy grail” of targeted therapies in autoimmunity, however, is the specific targeting of antigen-specific, pathogenic cells without the depletion of protective immunity. For this purpose, chimeric autoantibody receptors (CAARs) are being developed, whereby CAAR cells express the autoantigen on their surfaces and deploy cytotoxic effector functions against antigen-specific cells that bind to the autoantigen.^10^

Cellular therapeutics for autoimmune diseases are only now dawning and are likely to significantly reduce morbidity and mortality among patients with chronic disease, at least those who can afford them. New innovations are therefore urgently needed to make cellular therapies for autoimmune diseases affordable and accessible (e.g., in off-the-shelf approaches or *in vivo* CAR T cell generation).
